# "It's me who supports. How are you going to refuse to have a child?”: the social norms and gender dynamics of men’s engagement in family planning practices in the Democratic Republic of the Congo

**DOI:** 10.1186/s12978-025-02029-7

**Published:** 2025-06-27

**Authors:** Salomine Ekambi, Kathryn Sugg, Florence Mpata, Dédé Marachto Aliango, Peter J. Winch

**Affiliations:** 1 Johns Hopkins Bloomberg School of Public Health, Baltimore, MD USA; 2 CRIDESS-Université Protestante Au Congo CD, Kinshasa, Democratic Republic of Congo

**Keywords:** Social norms, Family planning, Birth spacing, Family size, Decision-making, Couple-communication, Modern contraception, Democratic Republic of Congo

## Abstract

**Background:**

In the Democratic Republic of the Congo (DRC), a disparity exists in the fertility desires of men and women, with men often expressing a desire for more children than their partners. This disconnect can be attributed to social and gender norms that influence discussions and decision-making regarding birth spacing, birth limiting, and the adoption of modern contraceptive methods. This qualitative study, through semi-structured interviews and focus group discussions, explored the social norms shaping perceptions, attitudes, and decision-making around family planning among men in the DRC.

**Methods:**

The study protocol was adapted from the 5-step process set forth in the Social Norms Exploration Tool (SNET). Data collection took place in the three provinces of Kasai Central, Lualaba, and Sankuru. This process was divided into three phases: a reference-group identification phase incorporating a short, quantitative questionnaire, an exploration of norms and attitudes with the population of interest (*N* = 317) – here married and unmarried men – and further exploration of social norms among the reference groups (*N* = 144) cited by men.

**Results:**

Social norms around family planning are contradictory and can be better understood by breaking down the concept of family planning into three categories of descriptive and injunctive norms: 1) the use of modern contraceptive methods, 2) birth spacing and family size, and 3) couple communication and decision-making. We found that while social norms oppose the use of modern contraceptive methods and advocate for larger family size, there is notable social support for birth spacing. Some men reported they would support their wives in learning about contraceptive methods if they were able to make the final decision. However, other men felt that allowing their wives to seek a method would undermine their authority or their virility.

**Conclusions:**

To increase modern contraceptive uptake, interventions should address the underlying issues that contribute to non-adherence, addressing the three categories and their associated norms individually and engaging reference groups important to each, including healthcare providers, religious leaders, and male peer groups, in family planning programming.

## Introduction/Background

Across sub-Saharan Africa, modern contraceptive method (MCM) prevalence is estimated at 23.4%, up 6.4% from 2012 [1]. However, MCM prevalence is much lower in the Democratic Republic of the Congo (DRC), with only 10.7% of married women on a modern method as of 2024, compared to 7.8% of married women in 2013 [2, 3]. Concurrently, 31.6% of married women surveyed for the 2023–2024 Demographic and Health Survey (DHS) expressed an unmet need for family planning, stating that they did not want to become pregnant at the time of the survey but were not taking a modern method to prevent pregnancy [2]. Reasons for non-use of a modern method include lack of access, as 70% of facilities offer MCM or counseling and only half of those facilities had all MCM in stock the day of the survey [4]. In addition, while the majority of the population (88% of women and 95% of men) had heard of at least one MCM, only 13% of women and 27% of men had heard something about family planning (FP) in the media, and 90% of women had not received any communication from a community health worker or health provider about family planning in the 12 months prior to the 2013–2014 DHS, suggesting that lack of knowledge around different methods and how to obtain them has historically been a barrier [3]. Finally, fear of side effects has also been shown to discourage women from adopting modern contraceptive methods [5].

Social norms may also constitute a barrier to contraceptive uptake. Social norms are the tacit rules of behavior shared by members of a community. These norms can be descriptive or injunctive, with descriptive norms illustrating what behavior is normal for members of a community and injunctive norms referring to behavior that is deemed appropriate or approved by others [6]. A distinction must also be made between collective norms and perceived norms – collective norms are the prevailing prescriptions and proscriptions for how a group behaves, while perceived norms are the individual interpretations of this code of conduct [7]. In this study, we are looking at perceived norms. The Theory of Normative Social Behavior posits that behavior is modulated by injunctive norms, outcome expectancies, and group identity [8]. In this study, we explored the concept of outcome expectancies as either the perceived benefits of a behavior or the perceived risk of breaking with social norms or socially normative behavior. The members of a community to whom an individual looks to understand the behaviors that are normative and acceptable or approved by others are considered “reference groups” for the behavior in question. Social norms have been shown to affect contraceptive use among both women and men [9]. A review of 13 qualitative studies found that the support of friends, family, and other network members (reference groups) was an enabling factor in contraceptive uptake among young people [10]. This finding highlights the importance of studying the effect of social norms on male engagement in discussions and decision-making around family planning [11].

The influence of social norms has been theorized to operate in at least two distinct ways: through social influence and through social learning [6, 11]. Social influence relates to the power of prestigious individuals to influence behavior and the importance of social approval or disapproval [12]. In a social network study in Niger, men were asked to identify “alters”,” or people they trusted with personal and important decisions. Male participants who believed that their alter(s) would support them in listening to their wives’ fertility preferences were more likely to have used modern contraceptives and to report spousal communication around family planning. Perceived social support was more significant for behavior than the actual attitudes of the alters towards family planning, showing the effect of social influence from close male peers [13]. Other studies in Ghana and Malawi have also found that network encouragement from male peers to use a contraceptive method is significantly associated with spousal communication around family planning, which in turn is positively associated with use of a contraception method by the couple [14]. These studies also show that the perception of whether or not other men were using a MCM (via men’s own assessment of the number of children and the number of years between their peer’s children) was influential for an individual’s use of MCM [15]. It is evident that both descriptive norms around whether other members of the community are using MCM, as well as injunctive norms relating to social support and encouragement are factors in spousal communication and use of family planning [13–15].

Social learning, which refers to “the process by which persons obtain information to reduce uncertainty in decision-making” [12], also plays a role in influencing MCM uptake. In studies looking at women’s social networks, women who were involved in credit groups were more likely to use a modern contraceptive method, in part (the authors hypothesize) due to exposure to innovative ideas from a heterogeneous group of women [16]. While women were more likely to explicitly discuss family planning, benefitting more from social learning than the social influence of observing other men’s family size and child spacing, men also discussed some of the benefits of birth spacing with male peers in a study in Malawi [15]. Finally, a study in Ghana found that interventions around family planning can spark informal network conversations among men that could contribute to social learning [14]. Observing other men and discussing experiences of family planning and birth spacing can contribute to how couples think and feel on the matter.

The social and gender norms that govern discussion and decision-making around birth spacing, birth limiting, and modern contraceptive use constitute a barrier to uptake of MCM. In one study in the DRC, less than half of men surveyed (46%) said that they approved of contraception, with another quarter saying they did not know how they felt about contraception [17]. Given that gender norms position men as decision-makers, lack of spousal communication around FP acts as a barrier to women’s access to FP services [5, 18–21]. Women who want to adopt a modern method may be unable to do so without prior discussion and approval from their male partner. Being cognizant of the power dynamics that limit women’s access to contraception requires incorporating a gendered perspective [21]. It is therefore the purpose of the analysis to look specifically at the social norms that govern men’s involvement in FP, and how it may contribute to women’s ability to adopt a contraceptive method.

In addition to investigating which factors, including social influence and social learning, influence men’s attitudes and behaviors, we discuss how the different descriptive and injunctive norms around “family planning” writ large are better understood when examined in relation to three sets of behaviors: (1) obtention and use of modern contraceptive methods, (2) birth spacing and birth limiting to achieve reproductive goals, and (3) communication and decision-making on FP within the couple. When we talk about family planning, we are talking about decisions and actions around the number and timing of children. However, the norms around the timing or spacing of births may be different from the norms around using a modern contraceptive method. Breaking down the overarching concept of family planning into its component behaviors and isolating social norms at the community level, as well as factors at the individual and structural levels, for each type of behavior will allow for more specific recommendations for the development of future interventions to increase modern contraceptive uptake and promote healthy birth spacing.

### Objectives/Research questions for this study

This study aimed to identify the social factors that shape men's perspectives, attitudes and involvement in the family planning process, shedding light on the influential figures and groups that contribute to and reinforce these norms in the three provinces of Kasai Central, Lualaba, and Sankuru in the DRC.

## Methods

### Study design

The study protocol was adapted from the 5-step process set forth in the Social Norms Exploration Tool (SNET) developed by the Passages Project [22]. The first step consisted of planning and protocol development, while the last two steps in the SNET provide guidance for analysis and use of results. Steps 2 and 3, identification of reference groups and exploration of social norms, guided the design of the present study. Reference groups, or those people in the community who influence men’s behavior around family planning, were identified through a short, tablet-based questionnaire in the first phase of data collection. Social norms were explored via individual interviews with married and unmarried men using a semi-structured interview guide (phase 2) as well as through focus group discussions (FGD) with members of the reference groups identified in the first phase of data collection (phase 3). Focus groups were facilitated by one data collector following a discussion guide, while another data collector took notes and monitored the audio recording device.

### Study area

The study was conducted in 12 health zones across three provinces in the DRC: Sankuru (Kabondo, Alengo, Dimoya, and Nyeme), Kasai Central (Mobutu, Kamupongo, Tshikumpele, and Matamba), and Lualaba (Biashara, Manika Marché, Nseke, and Kakanda). In each of the three provinces, one rural and one urban or peri-urban health district within a day’s drive of each other were selected for data collection, as there are differences in health center accessibility in urban areas compared to rural areas. In each health district, two health zones were selected to ensure greater variability in participant characteristics including language, ethnicity and geographic accessibility.

### Study population

The population of interest was married and unmarried men aged 15 −39 from 12 health zones. Participants were interviewed at their home on the day data collectors were present in the community. A total of 317 men from randomly selected households completed the tablet-based questionnaire, with every fifth household selecting an unmarried man and others recruiting married men. Additionally, 24 of the recruited men were selected for semi-structured interviews to provide deeper insights into family planning experiences. Men who had completed phase 1 were systematically invited to participate in the semi-structured interview until the desired number of married and unmarried men had been reached in each health zone.

FGDs were conducted based on the types of community members cited in the questionnaire. Male peers (8 FGDs) included married and unmarried men (15–39) who had not participated in the semi-structured interviews, while female partners (4 FGDs) comprised married women 15–39 years old. FGDs were also conducted with nurses (6 FGDs) and doctors (4 FGDs) who had worked at a public health facility in their health zone for at least one year. While healthcare providers were often the most cited group, there was some debate as to whether they constituted a “reference group” or were better characterized as a “source of information”. However, they were included as they may maintain and be subject to the social dynamics of the community, as well as having specific knowledge of the processes involved in seeking a contraceptive method. Finally, two FGDs were conducted with leaders of different religious groups in one rural health zone in Sankuru.

### Data collection

Data collection took place from March 11th to April 1st, 2022. Data collection was divided into three phases: a reference-group identification phase incorporating short, quantitative questionnaires, an exploration of norms and attitudes with the population of interest (married and unmarried men) and further exploration of social norms among the reference groups cited by men during Phase 1 (see Table 1).

**Table 1:** Data collection methods

Phase	Data collection Method	Objective	Population	N
1	Rapid, tablet-based questionnaire	Identify reference groups who influence men around family planning	Married and unmarried men 15–39 years and 18–39 years old, respectively	317
2	Semi-structured, face-to-face interviews	Examine how social norms affect male involvement in family planning; Examine men’s experiences with family planning, their involvement in the family-planning process, and their perception(s) of the male role in this process	Married and unmarried men 15–39 years and 18–39 years old, respectively	24
3	Focus group discussions with identified reference groups (24 FGD total)	Understand how reference groups communicate their attitudes, beliefs, and behaviors about family planning to the men they influence	Men between the ages of 15 and 39 years (friends of the population of interest) (8 FGD) Women between the ages of 15 and 39 years (wives of the population of interest) (4 FGD) Nurses (6 FGD) Doctors (4 FGD) Religious leaders (2 FGD)	144

Data were collected in the language in which the participants were most comfortable communicating, whether French, Swahili, or Tshiluba. Nine data collectors from the local firm ALMA Research Services were recruited for their experience with qualitative data collection and fluency in at least two of the languages cited above. All data collectors participated in a 5-day training on human subjects’ research and ethics and a module on Covid-19 transmission precautions.

In the first phase of data collection, eligible men who consented to participate were asked with whom they were most comfortable discussing family planning (regardless of prior opportunities for discussion). Using a tablet-based questionnaire, interviewers then asked follow-up questions to identify specific groups to whom they were comfortable talking.

In Phase 2, a subset of men from Phase 1 were interviewed on the same day they completed the initial questionnaire. Twenty-four men (six married and two unmarried men per province) who had completed the questionnaire were asked to participate in a semi-structured in-depth interview (IDI) of up to 90 min. This interview explored men’s knowledge, attitudes and perceived roles in family planning. The interview guide also included a vignette, presenting a fictional couple navigating discussions and decisions around using modern methods for birth spacing, was included to elicit responses about norms and community approval.

Phase 3 of data collection took place approximately a week after the initial phase, allowing the research team to analyze the data and identify two reference groups per site–-those most frequently cited by men in Phase 1. Community health workers were trained in recruiting focus group members who fit the reference group criteria (Table 1). Focus group discussions (FGDs) were designed to better understand men’s role in family planning, social norms shaping engagement, and key community influencers. Participants included friends, wives, nurses, doctors, and religious leaders. Discussions included open-ended questions, a shorter version of the vignette from Phase 2 IDIs and the “Five Whys” exercise, a root cause analysis where participants answered an overarching question followed by five consecutive “why” questions. Participants in focus groups were divided into subgroups and asked two initial questions. The second question that all focus group participants were asked was the same: “When a couple has decided to use family planning, some men do not allow their wife or partner to choose freely or independently which family planning method to adopt. Why is this?” In each province, one urban and one rural site was selected. Two focus groups were conducted with each of two reference groups per site, for a total of eight focus groups per province and 24 focus groups in total. 144 participants were included in the reference group discussions.

### Analysis

A 5-day participatory data analysis workshop of 14 participants was held with two Breakthrough ACTION-DRC program staff, three data collectors and the research coordinator from ALMA Research Services, and other stakeholders including six Ministry of Health representatives from the National Reproductive Health Program and two representatives of other partner and civil society organizations. This workshop served to validate initial themes arising from the transcripts and provide preliminary findings and program recommendations.

The initial themes discussed in the data analysis workshop were:

1. Norms around birth spacing
2. Knowledge of family planning methods
3. Benefits of modern contraceptive methods and concerns about modern contraceptive methods (split into two themes based on feedback from workshop participants)
4. Gender norms
5. Reference groups and sources of information (split into two themes based on feedback from workshop participants)
6. Sanctions for those who act against social norms

After validation of the above themes, participants analyzed the data according to this overarching framework, generating subthemes and classifying citations by theme as they went. Pairs of participants were assigned to the same geography, i.e. rural Sankuru, in order to discuss and validate their partner’s analysis.

A secondary analysis was conducted with an analytic questionnaire created in Google Forms to extract data from each of the transcripts (translated into French) according to a series of preset questions. For each question, a space was provided to enter exemplary citations. A separate analytic questionnaire form was designed for individual interviews with men and for focus group discussions with reference group members. This process of applying an analytic questionnaire to categorize and provide examples of different types of norms for each study participant, and identify the factors associated with each one, allowed more specific and detailed examination of the qualitative data.

## Results

The first phase of data collection allowed the researchers to identify which reference groups to include for the focus group discussions. Distinct types of influential community members, or "reference groups" reinforce the social norms affecting men’s behavior (see Table 2).

**Table 2:** Community members with whom men feel comfortable discussing family planning, by province

	**Reference group cited—% (n)**					
	Family member	Friends/peers	Community or religious leader	Healthcare provider	Other/do not know	**Total**
Kasai Central	24.8% (26)^b^	13.3% (14)	7.6% (8)	52.4% (55)^a^	1.9% (2)	**100% (105)**
Lualaba	25.7% (26)	37.6% (38)^a^	4% (4)	26.7% (27)^b^	6% (6)	**100% (101)**
Sankuru	10.8% (12)	21.6% (24)^b^	6.3% (7)	60.4% (67)^a^	0.9% (1)	**100% (111)**
**Total**	**20.2% (64)**	**24% (76)**	**6% (19)**	**47% (149)**	**2.8% (9)**	

^a^Top cited group in each province

^b^Second most cited group in each province

The two most influential groups are highlighted for each province. Healthcare providers (47%) were frequently cited as "community members with whom men feel most comfortable discussing family planning" due to their medical expertise. Male friends/peers (24%) and family members (20.2%) also played significant roles in shaping men's decisions regarding family planning. Additionally, while not prioritized as a group with whom men feel comfortable discussing family planning, religious leaders, namely “servants of God” (6%) came up as pivotal in forming normative beliefs.

The thematic analysis of the focus group discussions and interviews resulted in the identification of three distinct categories of social norms (see Table 3). These themes are categorized by both descriptive (practiced behaviors) and injunctive (expected practice) social norms that influence men’s perceptions, attitudes, and behavior.

**Table 3:** Three sets of family planning behaviors impacted by descriptive and injunctive social norms

	**Traditional versus modern methods:** Most couples practicing family planning use traditional methods of contraception and may not be motivated to adopt modern methods due to community disapproval	**Birth spacing and family size:** Couples practice birth spacing for economic and health reasons. Large families are common and approved	**Couple communication and decision-making:** Family planning is seen as a woman’s concern, but men are the decision makers in the household
**Descriptive norms** (beliefs about what behaviors most men in the community practice)	Men who use family planning methods are the minority while those who do not use are the majority; Men are aware of the different family planning methods that exist in theory but not in practice; Family planning is more of concern to the woman; the knowledge men may possess on the subject matter is relative to what the woman knows; Perception among men that if a woman were on a contraceptive, it would promote both prostitution and infidelity	Men look into FP when they are not in a position (financially) to produce children; when they become more financially stable having children becomes more of a priority; Men like and want many children even if their income is insufficient to support that number of children; Birth spacing is a white man’s/western concept; Men want to have many children; they believe that children are wealth	Decisions surrounding seeking information FP and obtaining contraception is at the couple’s discretion; not that of a third person; Although it may be common/necessary for the couple to consult a family member for counsel when the couple disagrees or have conflicting opinions, the man has the final decision; Men are often perceived as not requiring or actively pursuing information regarding family planning, largely due to the notion that it is the woman’s domain; FP is more of concern for woman (women bear the burden of reproduction)
**Injunctive norms** (beliefs about what behaviors others in the community will approve)	Contraceptives usage conflicts with religious beliefs and the messages of the church; Contraceptive is considered a sin Men who adhere to a FP are going against God; God wants mankind to “be fruitful and multiply”; To adhere to a contraceptive method as a man is to be perceived as powerless, sterile, and barren; Men risk losing their social status if they use or allow their wives to be on contraceptives; However, there are men in the community who believe that FP prevents diseases and allows parents to better raise their children	Having many births creates more harm than it does good; it is important to space the order of births by 2–3 years; Consistently producing children one right after the other is considered to be what animals do and should not be how humans reproduce; For those who do not use contraceptives, If a man and a woman marry, they must have children; it can be considered a sin if the couple does not multiply and reproduce; The family must be large, thus, if one child dies, there are other children in the family	The man is the head of the household and all decisions surrounding contraception and family planning must go through him; A woman must not make the decision on starting a contraceptive regimen without gaining permission from her husband; A man who seeks FP information may be criticized, and members of the community may say he is afraid of his wife

The first category, *family planning through modern and traditional contraceptive methods*, reflects hesitancy towards adopting modern methods and preference for more traditional methods. A considerable number of men displayed reluctance in utilizing modern contraceptives due to three primary reasons: 1) inadequate and limited awareness about modern contraception, 2) cultural and religious beliefs, and 3) social repercussions (stigma) associated with contraceptive adherence.

The second category, *birth spacing and family size*, refers to setting and pursuing goals regarding the amount of time between pregnancies and the number of children in the family. While birth spacing is widely recognized as essential for maintaining the health of both mothers and children, the term "family planning, "often associated with limiting family size, encounters resistance due to its perceived conflict with cultural and religious beliefs. On one hand, there is support for spacing births, and on the other hand, there is a desire for larger families, creating a complex dynamic of opinions and values. 

The third category, *Couple-communication and decision-making*, deals with questions of how couples discuss family planning, who initiates these conversations, and how the couple arrives at a final decision on whether or not to practice FP.

### Modern and Traditional Methods of Contraception

Family planning includes delaying childbearing, promoting birth spacing, and limiting family size through modern (e.g. condoms, implants, IUDs) and traditional methods. In both the focus group discussion and individual interviews, two categories of traditional family planning practices were commonly cited by participants: 1) absence of the husband/male partner during the postpartum period through travels to other communities/villages for an allotted time to avoid impregnating the woman and, 2) abstinence during fertile periods either through bed separation, or seeking other partners.

#### Descriptive norms

Participants noted that many men do not practice family planning through modern contraceptive methods. Descriptive norms suggest men favor (in practice and in theory) traditional methods.

*“P: another could travel so that he creates distance and the child can grow;*

*E: yeah, someone could go on a trip…*

*P: someone else could marry another woman, so that while the child is growing up, he is at another woman’s house;*

*E: ok, someone could marry another woman; another idea, what method...*

*P: Someone else could just stay and say ‘we are adults, let the child grow up’; he is there - he abstains but he is there”---- FGD with Partners/Spouses, Kasaï Central (R)*

For many men in the community, knowledge of risk, benefits, and availability of modern contraceptives is limited. Numerous respondents noted that despite being aware of a range of modern contraceptive options, men do not adopt these methods. Instead, men choose to leave the number of children they have up to God:

*“They know that the methods exist, they know, but most men don’t really know… how these methods work. So, in terms of, in terms of acceptability, or use of this service, it is still very low compared to women.” -- Doctors, Lualaba, (U)”*

*"They refuse, saying, ‘it’s God who gives me children. I must procreate as much as possible’”. – unmarried man, Lualaba, (U)*

Some participants admitted that hesitation to adopt modern contraceptives results from concern that these methods promote prostitution and infidelity among women. The rationale, as explained by many interviewees, is that the adoption of contraception methods prevents pregnancies, thereby facilitating women's covert engagement in promiscuous activities.

*“He has to know that the methods encourage women to engage in prostitution, that is to say that for the man when the woman takes a method, for him the woman will no longer be afraid of engaging in prostitution” - friends, Sankuru (U)*

Limited knowledge of modern contraceptives coupled with fear of the consequences that can arise from contraceptive adoption (I.e., infidelity, prostitution) influences decisions surrounding its use among couples.

#### Injunctive norms

Participants cited religious beliefs, sustained by religious institutions and their leaders. Religion, as cited by most participants, objects to the use of contraception, often portraying the practice as sinful. Men in the community shared that contraceptive use directly conflicts with the messages of the church which encourage mankind to be “fruitful and multiply”.

*“It is because you have stopped what God created, they want to destroy what God willed, you have organized yourselves to destroy the seed [fertilization], you are real sorcerers” -unmarried man, SAN (R)*

For many men, to adopt a contraceptive method is to risk being perceived as “powerless” and “sterile.” By permitting their spouses to engage in contraceptive practices, men expose themselves to potential criticism and a decreased social standing.

*“People judge you based on the children you have in the world and you come with your practice [family planning] of having children like this, like that, they will say this, you are sterile or impotent” -unmarried man, Sankuru (R)*

*“For Catholics, condoms are rejected... according to the information I get from them, anyone who uses a condom is like somebody who's had an abortion because they've thrown away the sperm.”- FGD with nurses, Sankuru (U)*

Messages about family planning from religious institutions, combined with the stigma attached to men adopting modern contraception, shape decision-making regarding modern contraceptive methods.

### Birth spacing & family size

The transcripts emphasize a broader understanding of the advantages of birth spacing and pursuing goals around family size more generally, beyond the benefits and potential harms of contraceptive methods generally and modern methods specifically. While birth spacing is seen as crucial for preserving maternal and child health, the phrase “family planning,” which is commonly understood as limiting family size, is frequently opposed due to it interfering with cultural and religious beliefs. There are conflicting norms regarding pursuing family planning goals, with support voiced for both spacing births and having large families. Those who practice birth spacing often reference healthcare providers who emphasize the medical advantages of spacing births and the risks of frequent childbirth or male peers whose wives have experienced unfavorable birth outcomes. However, the promotion of larger family sizes is commonly advocated by religious institutions/leaders, along with encouragement from family, male peers, and friends, who urge men to “add to the Earth”.

#### Descriptive norms

Numerous respondents revealed the tendency among men to embrace birth spacing or limiting when they are unable to financially support a large family. Conversely, having more resources incentivizes them to desire more children. Nevertheless, it is observed that many men have a desire to have as many children as feasible, even when they lack the resources to sustain a sizable family. In response to family size expectations, respondents frequently stated that men perceive that,"children are wealth". A spouse in a focus group stated:

*"Men look [at birth spacing] when they don't have means. That's when they say: no, we don't have money, we have to pace ourselves with the births. That's how they are. When he earns a little money, he will tell you, ‘it's me who supports. How are you going to refuse to have a child?’"---- Partners/Spouses, Lualaba, (R)*

Another participant expresses that a large family size is highly desirable amongst men in the community:

*“No, because I know the habits here at home very well, people like to have a lot of children, men like to have a lot of children, I don’t know if it’s honor or what.”– Married man, Sankuru, (U)*

Having children is a priority for many in the community, regardless of the financial capabilities to support an increased family size.

#### Injunctive norms

Participants referenced that men who adhere to family planning and birth spacing concepts recognize that it is advantageous in protecting the family. These men frequently cite messages of health personnel on how birth spacing significantly reduces the disease burden for mother and child thus lessening the financial burden of having to care for sick children. Birth spacing does not discourage the desire for a sizeable family unit; instead, it is said to promote peace and health in the family home. A married man explains:

*“When you are a responsible man, you know only that I must protect my home. As soon as I have a child... I am going to protect him... even the doctors speak, even on radios, giving this advice that it is good to leave certain [birth] spaces."-Married man, KC (U)*

*“They talk about it, that in our country, there are children who often burden the parents, these children can die from events [sicknesses] like that, when you plan [your births] this allows you to raise the children correctly, and spare the children many illnesses. Then the parents will be able to raise these children"-*-----Friends, Sankuru, (U)

Religious beliefs also play a significant role when it comes to birth spacing and family size discussions. Numerous participants reported that there are men in the community who perceived having children as a religious obligation. Men must “be fruitful and multiply” otherwise they are defying the will of God.

*“We see it as if it were a sin, if someone does not have children it's like a sin.” – married man, Sankuru (U)*

The perspective of family members and male peers significantly influences a man's determination of family size. When men gather to converse about family planning, two main types of discussions transpire. One type involves sharing personal struggles and experiences, cautioning others about the risks of disregarding birth spacing guidelines. The second type dissuades men from adopting contraceptive methods, instead urging them to continue expanding their families. A discussion between male peers on one's own challenges might proceed like this:

*"He asks his friend, ‘Is that your baby?'. He answers ‘yes, it's my baby'. ‘Their mom isn't pregnant?' ‘No, she's not pregnant.' He looks at the baby like he's already grown up, he's going to ask his friend, ‘but how do you do it?’”-- Partners/Spouses, Lualaba, (R)*

Male peers who support large families can advise one another as follows:

*"My friend how? Are you married? How many children do you have? Ah! My friend, you must add [children]. The advantages ----perhaps he [the child] is a worker."- Friends, Lualaba (U)*

Religious belief is at the root of many decisions relating to family size with many in the community supporting a large family. However, some men see the benefits of birth spacing, citing the health benefits associated with this concept. The influences of family and peer groups can also sway decisions on birth spacing and family size.

### Couple communication & Decision-making

Couple communication and decision-making refer to how a couple decides who seeks information on FP and the choice of contraceptive. When a couple has conflicting views on the subject it is not uncommon for a couple to consult a third party. Health experts, religious leaders, and godparents/elders within the family were frequently mentioned when participants were asked about external parties that can be involved in a couple’s family planning decisions. Health professionals were referenced due to their medical training, while community elders were noted for their familiarity with the couple and family upbringing experience. Pastors were cited for their theological expertise. These reference groups were chosen because of their potential to facilitate couple decision-making in the realm of family planning methods and family size considerations.

#### Descriptive norms

Many participants concurred that when a couple is torn about family planning and the decision to increase or limit family size, it may be necessary for them to seek a third opinion. Men might find it more comfortable to seek advice from their peers, while couples could turn to godparents or community elders for guidance when making decisions. However, the consensus is that it is ultimately up to the couple to decide which of them should seek out information on family planning and where. As stated below:

*“A third party does not have the right to impose themself on the decision of a couple...the final decision comes down to them both.” --Married man, Sankuru (U)*

Participants also admitted that women are more knowledgeable about family planning while men lack information. It is commonly believed that women are responsible for procreation; hence it is their duty to be more informed about available contraception. During one focus group discussion, it was stated:

*“The reasons that have been put forward are as follows: some men lack information, and it is women who are more interested in family planning or FP methods.... men do not consult providers”* ----Doctors, Lualaba, (U)

#### Injunctive norms

Participants reasoned that because the man is the head of the household, he must make all decisions on family planning and choice of contraceptives, if any. It is socially unacceptable for a woman to decide to get a contraceptive method without first obtaining her husband's approval. The woman is “behind the man" and thus the man makes all the final decisions on the subject matter. It is said:

*“It's bad because I'm the husband who is also the head of the family, you must tell me, and I must give permission... you leave me, the head of the house, to go alone [to seek contraception] without my permission, it is an aberration; this way of doing things is bad. It is an aberration."-unmarried, Sankuru (R)*

Several participants noted that although some men seek family planning information, these men bear the risk of criticism from other community members. These may be accused of being fearful and controlled by their wives.

*“He has already been dominated by his wife. He's the one who should be making a decision about that."– unmarried man, Lualaba (U)*

While a couple may solicit input from an external party to influence their family planning decision, the ultimate responsibility lies with the couple themselves. Nevertheless, within the partnership, regardless of which individual possesses greater contraceptive knowledge, men retain the authority to finalize all decisions. Furthermore, men who opt for contraception and adhere to it may face possible criticism and stigma.

**Fig. 1:**
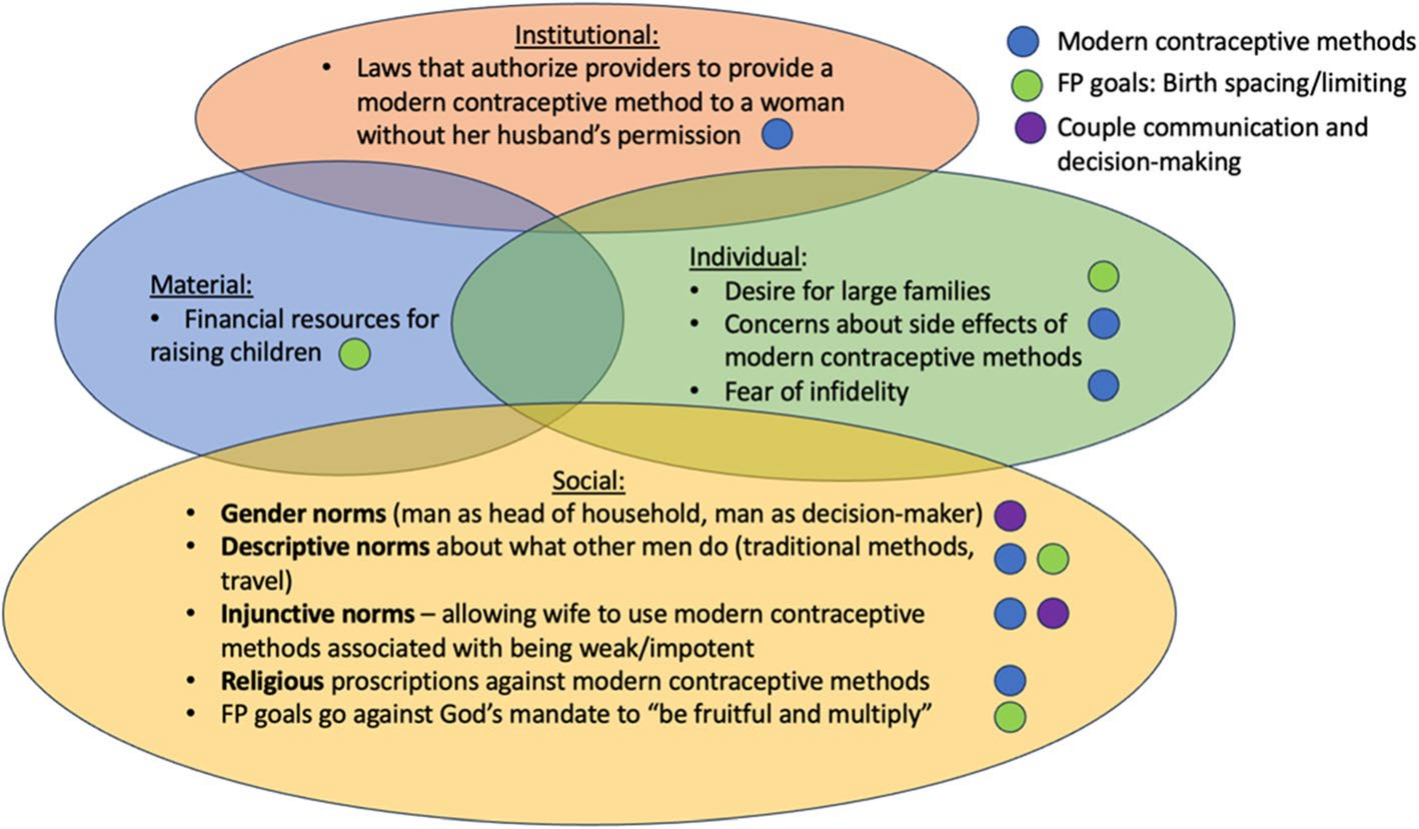
Socio-ecological model for power and gender influence on family planning behaviors, building on framework of Cislaghi & Heise (2019)

This qualitative study unveiled broader impacts of social norms and network effects, which shape perception, attitudes, and behaviors on decision-making around family planning in the Democratic Republic of the Congo (Fig. 1).

By analyzing descriptive and injunctive social norms that contextualize participants' behavior, we identified three categories of norms: 1) family planning using both traditional and modern contraceptives, 2) birth spacing and family size, and 3) couple communication and decision-making (see Table 4). Within each category, reference groups or "norm setters" shape perceived descriptive and injunctive norms.

**Table 4:** Three categories of social norms – Extended version incorporating framework from Cislaghi & Heise (2019) [23]

Factors	**Family planning/methods**	**Birth spacing/family size**	**Couple communication and decision-making**
**Individual factors**	Knowledge, skills, self-efficacy related to contraception Male and female education	Aspirations Attitudes Religiosity	Women’s empowerment Male and female education
**Social factors**	Rumors about contraceptive side effects	Social networks Household and kin group structures and support Positive deviants	Power relations Gender norms
**Material & institutional factors**	Method mix offered in public health services and in private sector Marketing of contraceptives Fees for services, cost of contraceptives	Financial implications of larger & smaller families Cost and availability of schools Wealth, sources of income	Laws – family inheritance, property, women’s rights
	
**Descriptive social norms**	Perception of which methods others in the community use	Perception of birth spacing practices and family size of others in the community	Perception of how others make decisions about family planning, and about how others seek FP information and services
**Injunctive social norms**	Perception of which methods others approve and disapprove	Perception of the birth interval and family sizes that others approve and disapprove	Perception of ways of making decisions and seeking FP information and services that others approve and disapprove

## Discussion

One key finding, in line with other studies, is participants’ attitudes and preference for traditional over modern contraception [24, 25]. Focus group discussions and interviews revealed that this preference was largely due to limited knowledge and misconceptions on the risk and benefits of modern contraceptive methods. A systematic review in sub-Saharan Africa found that misinformation, including fears that contraception promotes infidelity, reduces uptake [24]. Our study further uncovered that social factors such as rumors about possible contraceptive side-effects like infertility further alienate couples from adhering to modern methods. Many women view childbearing as crucial for earning their husband's respect, stabilizing relationships, and ensuring economic support, making infertility a significant social and economic threat [24].

A Kenyan study reported that when young men believe misconceptions linking contraception to infertility, their willingness to utilize modern methods is significantly reduced [26]. This finding highlights the need for targeted health education, specifically among male peer groups. In Malawi, male outreach workers successfully promoted contraceptive uptake through community dialogues [27]. Mentorship programs with community health workers can improve contraceptive access by addressing side effects and couples’ concerns during community visits [28].

Participants in our study supported birth spacing but were less favorable to family planning if it meant limiting family size. While birth spacing was seen as beneficial for promoting maternal and infant wellbeing, the use of modern contraceptives threatens the desire and, in some contexts, the religious mandate to produce more children. Despite the financial implications of a larger family, members in the community believe that “children are wealth”. Existing literature confirms that birth spacing is more widely accepted whereas limiting family size challenges social-cultural norms that value large family size [28]. Rather than endorsing "family planning, "we suggest advocating for "birth spacing", a concept that supports individuals in achieving their objective of maintaining healthy spacing between pregnancies, as it better aligns with community norms. Male champions can play a key role in promoting the health, financial and educational advantages of birth spacing [28].

As highlighted in our findings, community members perceive children as a valuable resource and birth spacing allows for the desired family size while protecting maternal and child health [5, 29, 30]. Religious leaders can also play a vital role in mobilizing interventions. Our study revealed that religious leaders and messages from religious institutions influence decision-making and perceptions about contraceptive use and family size. This result aligns with other research showing that religious condemnation of contraceptives is linked to underutilization of family planning services [9, 26]. Engaging religious leaders in reviewing religious texts that support birth spacing and limiting can be instrumental in shifting community perceptions [28]. Additionally, evidence shows that mass-media communication programs are effective in promoting family planning and influencing reproductive behavior [31–33]. For example, a Nigerian study using radio serial drama as a communication tool demonstrated a positive impact on reproductive outcomes and addressed concerns about contraceptives being “against the will of Allah” [33].

In our third category, *couple communication and decision-making,* gendered power dynamics were identified. While couples may seek external input for family planning disagreements, the final decision often rests with the male partner as the household head. Even when women are more knowledgeable about contraceptive methods, social norms discourage independence, requiring them to seek their husband's approval. This finding aligns with previous literature, where researchers found that the decision-making authority of husbands negatively influenced women's contraceptive intentions in sub-Saharan Africa [34, 35].

A study of church-going young men in Kinshasa, DRC, found that while 92% believed couples should decide together on having children, 94% felt men should have the final say, and 79% saw pregnancy prevention as the woman’s responsibility [36]. These beliefs may hinder couple communication and shared decision-making. To address this barrier, programs should actively involve men through community dialogues with male outreach workers [27]. A DRC study demonstrated that men who are more involved in maternal healthcare were more likely to discuss postpartum FP with their partners [37]. Strengthening couple communication around birth spacing requires involving men throughout the pregnancy, delivery, and neonatal care, fostering better understanding of reproductive and contraceptive uptake.

Both injunctive and descriptive norms are shaped by social networks, with perceived contraceptive use among peers influencing individual adoption more than actual use [13]. Similarly, in this study, male friends and peers played a key role in promoting and/or discouraging the use of family planning services. When a couple faces uncertainty — such as when the woman wants to adopt a contraceptive method but the man refuses—descriptive norms suggest that it is socially acceptable to consult a trusted outside source. The third party engaged in the couple's decision-making can significantly sway the man's viewpoint.

A study on social network influence on FP behaviors in Nigeria indicated that male respondents who believed their social network would endorse their receptiveness to their spouse’s fertility preferences were more likely to be engaged with family planning services [13]. This further highlights the influence of male peers in contraceptive decision making. Participants of the present study in DRC also disclosed that men risk being criticized for engaging with family planning services. While an individual might gain knowledge or a change in attitude through an individual intervention, fear of social disapproval will remain unless others in their social network also participate. Engaging reference groups such as male peer groups, health professionals, and religious leaders could render interventions more effective in increasing uptake of modern contraceptives.

Given that birth spacing is already an accepted practice, future interventions should prioritize promoting modern contraceptive methods as a tool for effective spacing. Interventions should engage men in familiar social settings, addressing social norms as well as knowledge and attitudes. Training male peer educators and elevating the voices of men with experience using contraceptive methods for birth spacing will be important to increasing trust in the messaging and in framing contraceptive uptake as relevant to the lives of men in the community. Research indicates that programs using a gender-transformative approach and promoting gender equity are more effective at changing behavior than those with a narrow focus [38]. By engaging key influencers, addressing social norms, and framing modern contraception as a tool for birth spacing, future interventions can foster greater acceptance and adoption of family planning, ultimately improving maternal and child health outcomes (see Table 5).

**Table 5:** Reference groups for family planning behaviors impacted by descriptive and injunctive social norms

	**Modern and traditional methods of contraception**	**Birth spacing and family size goals**	**Couple communication and decision-making**
Descriptive	• Health providers • Male peers	• Family members	• Family members • Male peers
Injunctive	• Religious leaders	• Religious leaders • Male peer groups • Health providers	• Male peers
Implications for intervention design	Engage men with their male peers. While an individual might profit from an intervention in terms of knowledge and attitudes, he will still be wary of others' disapproval unless he knows that they have been part of the same intervention and discussed the benefits of modern FP Providers should be mentored on addressing rumors and managing side effects of MCMs, as done with community health workers in Uganda [28]	Focus messaging on CBS (childbirth spacing) rather than “family planning” or “limiting” [28] Use male role models to promote the benefits of birth spacing (financial, education, health) to men [28] Increase risk perception of closely spaced births through mass media for men, women, and family members [33] Review aspects of religious texts (such as the bible) and highlight passages that aligned with approval or acceptance of birth spacing and/or limiting [28, 39]	Men/male peers should be the primary focus of any intervention on couple communication. Women may feel unable to start conversations around contraception, while men may not be thinking about FP/CBS or fear stigma around initiating these conversations. Using men to facilitate the community dialogues will help reach husbands, and the message may resonate more coming from a male peer. Similar interventions in Malawi and Kenya saw greater couple communication and support for FP, as well as increased contraceptive uptake in Malawi [27, 40]

### Study limitations

This study is not without its limitations. As qualitative research, findings from our study do not represent all community members and are not generalizable. Due to the interview questionnaire's framing, we may not have captured other key influential groups with whom people are not comfortable discussing family planning. Future social norms explorations should attempt to also identify the reference groups that men feel are most likely to disapprove of family planning. It is important to note that, as with any survey that explores sensitive topics related to gender equity and sexuality, despite assurances of participant anonymity, there is the possibility that some sensitive issues may have been either underreported or overreported.

## Conclusions and recommendations

This study delves into the varied descriptive and injunctive norms encompassing the broader concept of "family planning.” Deconstructing the overarching concept of family planning into three specific categories (use of traditional and modern methods; birth spacing and birth limiting to achieve reproductive goals; couple communication and decision-making on FP) and dissecting social norms at the community level enables more targeted recommendations for family planning interventions. To increase modern contraceptive uptake, interventions should address the underlying issues that contribute to non-adherence. Future programming should involve engaging influential groups such as healthcare providers, religious leaders, and male peer groups into family planning programming, with targeted interventions for each reference group.

### Recommendations for engaging health providers

- Engage health providers to talk to men, individually as well as in groups, about the benefits of contraception for postpartum family planning and birth spacing of two years or more (injunctive social norms: health providers are a powerful source of social influence).
- Train health providers in responding to rumors and misinformation about contraceptives in a clear and specific way.
- Train health providers on couples counseling and method choice, explaining the different options and their benefits and side-effects for couples in general and for postpartum women.
- Strengthen knowledge of laws around sexual and reproductive health that state that women do not need their husband’s permission to accept a contraceptive method.

### Recommendations for engaging religious leaders:

- Engage religious leaders to speak in favor of birth spacing as a means of improving the health of the mother, newborn, and family (it is not a sin to take effective action to protect the health of your family).
- Engage religious leaders to speak in favor of communication within the couple about birth spacing, reframing male authority as a responsibility to start conversations with his wife about how to protect her health and the health of their children.

### Recommendations for engaging men

- Engage men in community dialogues led by respected health providers to talk about the benefits of contraception for birth spacing and to debunk rumors.
- Train male peer educators to talk about family planning with other men, individually and in groups.
- Discuss the benefits of men supporting their wives and newborns by helping at home and using a modern method to space pregnancies rather than practicing abstinence by traveling for long periods after their wife has given birth.
- Encourage men to discuss birth spacing with their partner, including how long they want to wait in between children and which methods are available. Provide support in the form of role playing or a “cheat sheet” of helpful questions to start the discussion.
- Encourage men to accompany their partners to the health center to learn more about the different contraceptive methods.
- Reframe talking about birth spacing and contraception as the responsibility of the head of household to care for his family, including the health and wellbeing of his wife and children.

## Data Availability

No datasets were generated or analysed during the current study.

## Abbreviations

DRC: Democratic Republic of Congo
FGD: Focus Group Discussions
FP: Family Planning
IDI: In-depth Interviews
MCM: Modern Contraception Methods
(R): Rural
SNET: Social Norms Exploration Tool
(U): Urban
USAID: United States Agency for International Development
